# Bone Mineral Affinity of Polyphosphodiesters

**DOI:** 10.3390/molecules25030758

**Published:** 2020-02-10

**Authors:** Yasuhiko Iwasaki

**Affiliations:** Faculty of Chemistry, Materials and Bioengineering, Kansai University, 3-3-35 Yamate-cho, Suita-shi, Osaka 564-0836, Japan; yasu.bmt@kansai-u.ac.jp; Tel.: +81-6-6368-0090

**Keywords:** biomimetics, polyphosphodiester, ring-opening polymerization, biodegradable polymer, biomineralization, nanoparticles, bone targeting

## Abstract

Biomimetic molecular design is a promising approach for generating functional biomaterials such as cell membrane mimetic blood-compatible surfaces, mussel-inspired bioadhesives, and calcium phosphate cements for bone regeneration. Polyphosphoesters (PPEs) are candidate biomimetic polymer biomaterials that are of interest due to their biocompatibility, biodegradability, and structural similarity to nucleic acids. While studies on the synthesis of PPEs began in the 1970s, the scope of their use as biomaterials has increased in the last 20 years. One advantageous property of PPEs is their molecular diversity due to the presence of multivalent phosphorus in their backbones, which allows their physicochemical and biointerfacial properties to be easily controlled to produce the desired molecular platforms for functional biomaterials. Polyphosphodiesters (PPDEs) are analogs of PPEs that have recently attracted interest due to their strong affinity for biominerals. This review describes the fundamental properties of PPDEs and recent research in the field of macromolecular bone therapeutics.

## 1. Introduction

Polyphosphoesters (PPEs) are well known for their flame-retardant properties and consequently various synthetic procedures that use these polymers are currently being considered [1]. However, PPEs are also of great interest in biomedical fields due to their structural similarity to naturally occurring biomolecules [2,3,4,5]. PPEs can be synthesized by transesterification [6], ring-opening polymerization (ROP) [7], polycondensation [8], enzyme polymerization [9], and metathesis reaction [10], among which ROP is one of the most promising processes. ROP was mainly contrived by Penczek et al. [11], who synthesized PPEs from cyclic phosphoester monomers with five- or six-membered rings [12].

The use of both anionic and cationic ROP has been examined for PPE synthesis, but it has proven difficult to obtain high molecular weight PPEs with the cationic process. Consequently, in the early days, alkyl aluminum and metal alkoxide were used as catalysts for anionic ROP. However, while metal catalysts are useful and show high catalytic activity, they are atmospherically sensitive and it is difficult to control the polymerization process. ROP of cyclic phosphoesters has also been successfully achieved using organocatalysts, such as 1,8-diazabicyclo [5.4.0]undec-7-ene (DBU) or 1,5,7-triazabicyclo[4.4.0]dec-5-ene [13], which exhibit weak catalytic activity and consequently are easy to use and allow the ROP to proceed in a living manner, resulting in a very narrow molecular weight distribution (Mw/Mn < 1.1). Clément et al. [14] subsequently improved the polymerization condition by adding thiourea to the reaction system, allowing a narrow molecular weight distribution to be maintained with a high yield (>60%). Today, most studies use organocatalysts for the synthesis of PPEs through ROP.

**Figure 1:**
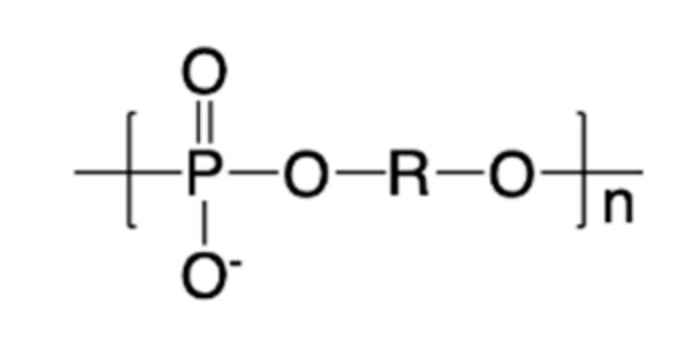
General structure of polyphosphodiesters (PPDEs).

The phosphorus in PPEs can form three stable and divergent bonds in addition to the P=O bond, which offers advantages over conventional biodegradable polymers such as aliphatic polyesters and polycarbonates and means that side-chain functionalization of the polymers is possible even if two bonds are used in formation of the polymer backbone. Moreover, end-functionalization of PPEs is also easily performed by changing the initiator or through post-polymerization modification. Polyphosphodiesters (PPDEs) are functionalized PPEs that have a similar structure to the backbone of nucleic acids (Figure 1) and are highly water-soluble, biodegradable, and biocompatible. It has also recently been shown that PPDEs have a high mineral affinity [15]. In this review, the fundamental properties of PPDEs and their therapeutic capabilities for bone treatment are described. 

## 2. Synthesis of Polyphosphodiesters (PPDEs)

**Figure 2:**
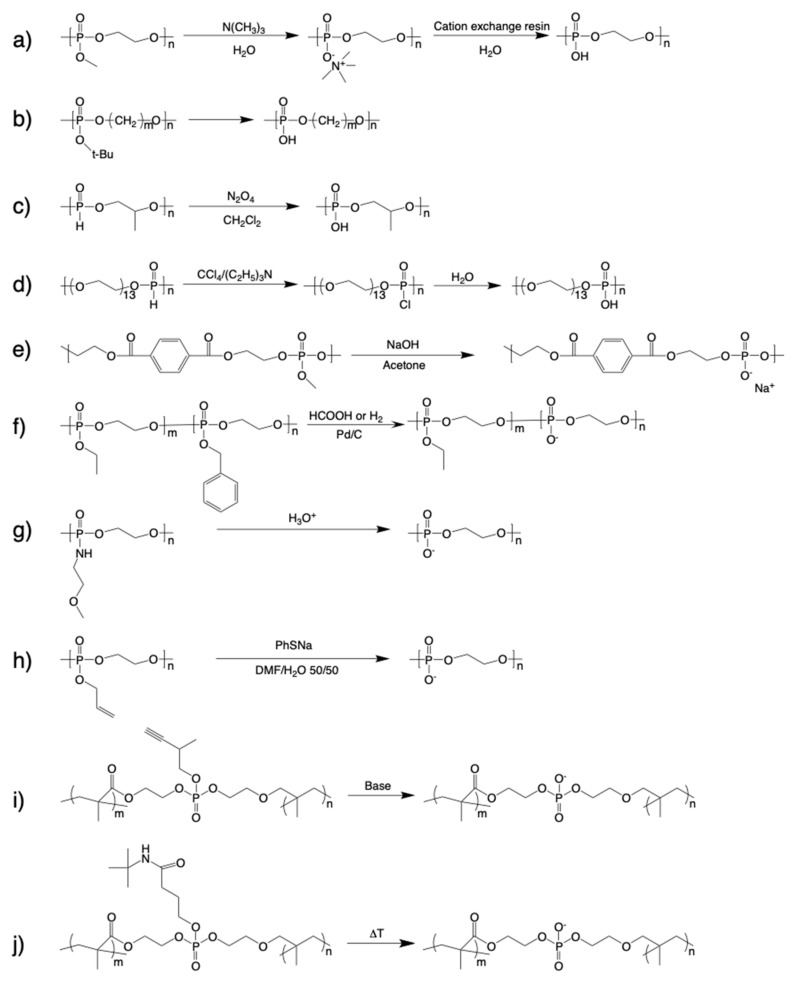
Synthetic routes to polyphosphodiesters (PPDEs).

The synthetic routes to polyphosphodiesters (PPDEs) are summarized in Figure 2. One of the earliest procedures was reported by Penczek et al. [16] and involved reaction of the neutral PPE poly(methyl ethylene phosphate) (PMEP) with trimethylamine in aqueous solution to produce a polysalt, which was then treated with a cation exchange resin to yield poly(ethylene phosphate) (PEP) (Figure 2a). This procedure resulted in a high degree of demethylation and no degradation of the polymer backbone. Alternatively, Yasuda et al. [17] reported the thermal elimination of isobutylene from PPEs bearing tert-butoxy groups (Figure 2b). However, cyclic monomers with tert-butoxy groups are very heat sensitive and cannot be purified using vacuum distillation. Poly(alkylene H-phosphonate)s can also be used to generate PPDEs through oxidation with nitrogen dioxide [18] or transformation via the Atherton–Todd reaction [19] (Figure 2c,d). PPDEs have also been produced through the demethylation of polyphosphotriesters, which involves treating the polymer with sodium hydroxide, as shown in Figure 2e [20]. However, there is a risk of backbone degradation occurring during this process because PPEs are easily degraded under basic conditions. Copolymers composed of phosphodiester and phosphotriester units have been synthesized by removing benzyl protecting groups on PPE precursors via hydrogenation (Figure 2f) [21]. However, while this process is clean and useful for controlling the molar fraction of phosphodiester and phosphotriester units, the purification of the monomer bearing the benzyl group is difficult due to the high boiling point. 

More recently, Wooley et al. [22] reported an alternate process for generating PPDEs that involved acid-assisted cleavage of the phosphoramidate bonds along the backbone of polyphosphoramidates (Figure 2g). However, while PPEs are relatively tolerant of acidic conditions compared with basic conditions, strong acidity (pH = 1) may be harmful to polymer chains. In contrast, the deprotection of poly(allyl ethylene phosphate) using sodium benzenethiolate to give poly(ethylene sodium phosphate) (PEP·Na) (Figure 2h) has proven quite successful because the allyl-substituted monomer 2-(prop-2-en-1-yloxy)-2-oxo-1,3,2-dioxaphospholane is easily purified by fractional distillation and the pendant allylic groups are selectively deprotected by a reaction with sodium benzenethiolate without any detectable degradation [23]. Finally, Dera et al. reported the preparation of PPDE crosslinkers from cyanoethyl-bearing phosphotriester precursors with sodium hydroxide (Figure 2i) [24]. They also reported an alternative thermal elimination process (Figure 2j) [25] to avoid the use of a base that may adversely affect the backbone structure, as mentioned above.

## 3. Mineral Affinity of Polyphosphodiesters (PPDEs)

**Figure 3:**
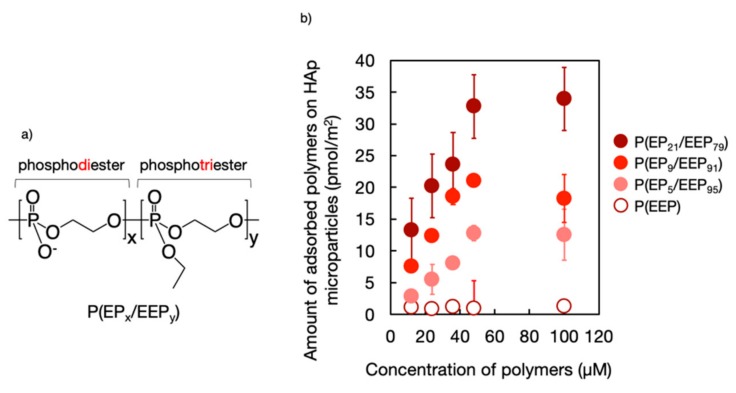
(**a**) Chemical structure of polyphosphoester copolymers (P(EP_x_/EEP_y_)) and (**b**) the effect of the phosphodiester composition of the copolymers on their mineral affinity.

To clarify how the chemical structure of PPEs affects their mineral affinity, copolymers (P(EP_x_/EEP_y_)) composed of phosphodiester, ethylene phosphate (EP) and phosphotriester, and ethyl ethylene phosphate (EEP) units (Figure 3a) were synthesized using the procedure, shown in Figure 2f, which involved the complete deprotection of the benzyl groups of the precursor [21]. It was found that the amount of copolymer that was adsorbed on hydroxyapatite (HAp) microparticles increased with an increase in the composition of EP units in the copolymers (Figure 3b) [15], indicating that the ionized phosphodiester unit was the key element exhibiting mineral affinity. Interestingly, it was also found that the copolymers could reduce HAp formation and resorption, which is similar to the phenomenon that was observed following the treatment of HAp with bisphosphonates, which are the first-line drugs for treating osteoporosis and adsorb remarkable amounts of polymers. 

The effect of PPDEs on biomineralization has also been investigated, both in aqueous solution and on a solid surface. Penczek and coworkers [26,27] synthesized the triblock copolymer poly(ethylene glycol)-poly(alkylene phosphate)-poly(ethylene glycol) and found that it modulated the crystallization of CaCO_3_ particles, resulting in the formation of unique features consisting of semi-open empty spheres that were composed of small crystallites with a diameter of 40–90 nm. Christine et al. also studied the templating capacity of PPDEs for the synthesis of CaCO_3_ particles [28]. 

**Figure 4:**
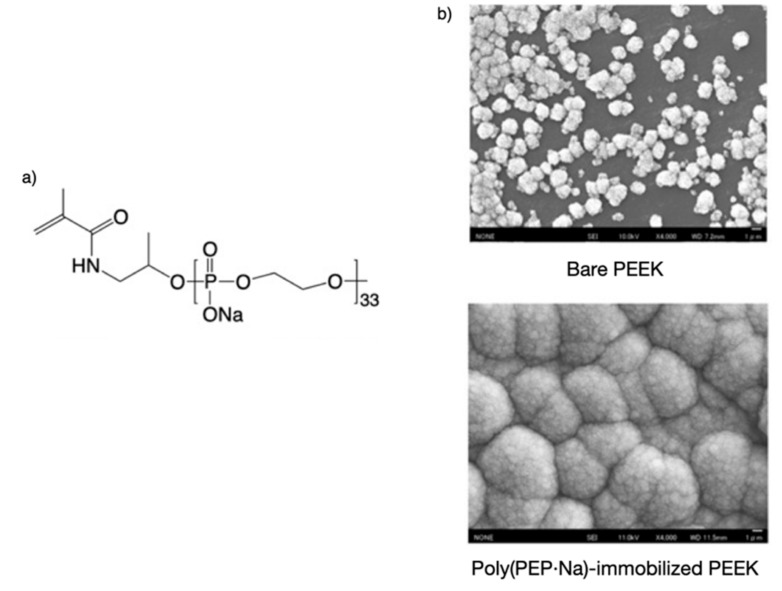
(**a**) Chemical structure of the polyphosphodiester PEP·Na macromonomer and (**b**) scanning electron micrographs of poly(ether ether ketone) (PEEK) and poly(PEP·Na)-immobilized PEEK specimens that had been soaked in ×1.5 simulated body fluid (1.5 SBF) for 28 days.

PEP·Na macromonomers have also been synthesized (Figure 4a) to immobilize poly(ether ether ketone) (PEEK) and thus enhance biomineralization on the surface of PEEK grafted with poly(PEP·Na) [29]. These macromonomers were synthesized via the demethylation of poly(m-phenylene) (PMP) macromonomers through the ROP of MP using 2-hydroxypropyl methacrylamide as an initiator. Although the synthesis of the macromonomer was tried by using 2-hydroxyethyl methacrylate as the initiator, the polymerizable group was cleaved during the demethylation process shown in Figure 2a. Surface modification of PEEK was then performed via photoinduced and self-initiated graft polymerization of the macromonomer. Figure 4b shows scanning electron micrographs of PEEK and poly(PEP·Na)-immobilized PEEK specimens that had been soaked in ×1.5 simulated body fluid (1.5 SBF) for 28 days. As can be seen, the surfaces of the poly(PEP·Na)-immobilized PEEK specimens were completely covered with spherical, cauliflower-like mineral deposits that resembled calcium phosphate crystals, whereas the surfaces of the PEEK specimens had few of these mineral deposits.

## 4. Polyphosphodiesters (PPDEs) in Mineral-Binding Nanoparticles

**Figure 5:**
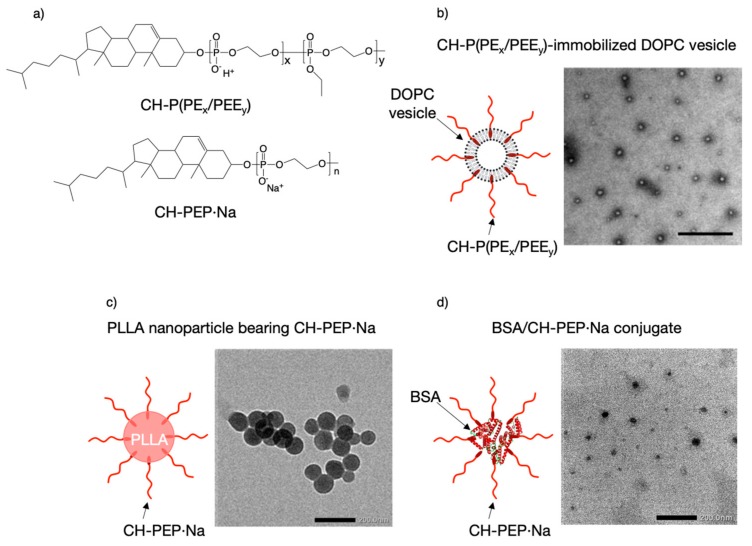
(**a**) Chemical structure of amphophilic PPDEs. (**b**) Amphiphilic polyphosphodiester copolymer [CH-P(EP_x_/EEP_y_)]-immobilized 1,2-dioleoyl-sn-glycero-3-phosphocholine (DOPC) vesicles. Scale bar represents 500 nm. (**c**) Poly L-lactic acid nanoparticles bearing amphiphilic poly(ethylene sodium phosphate) (CH-PEP·Na). Scale bar represents 200 nm. (**d**) Bovine serum albumin/CH-PEP·Na conjugates. Scale bar represents 200 nm.

As mentioned above, PPDEs show an excellent affinity for minerals. Consequently, several mineral-binding nanoparticles have been designed using amphiphilic PPDEs (Figure 5a) as surface protectors. Cholesterol (CH) is a major candidate as an initiator in the preparation of amphiphilic PPDEs because alcohols can be employed for organocatalytic ROP of cyclic phosphate monomers. Amphiphilic P(EP_x_/EEP_y_) [CH-P(EP_x_/EEP_y_)] that was composed of EP and EEP units was first synthesized and immobilized on 1,2-dioleoyl-sn-glycero-3-phosphocholine (DOPC) vesicles (Figure 5b), which resulted in the zeta-potential becoming more negative with increasing amounts of immobilized CH-P(EP_x_/EEP_y_) [30]. The stability of the vesicles was then effectively improved by surface modification and was controlled by changing the fraction of DOPC and CH-P(EP_x_/EEP_y_). The surface-modified vesicles were found to have a high cell compatibility at prescribed phospholipid concentrations and an improved affinity to calcium deposits generated by MC3T3-E1 cells.

Poly L-lactic acid (PLLA) nanoparticles bearing amphiphilic PEP·Na (CH-PEP·Na) have also been prepared using a solvent evaporation technique [31]. These nanoparticles had diameters of approximately 100 nm, exhibited high colloidal stability (Figure 5c), and did not show hemolysis activity or cytotoxicity against MC3T3-E1 cells. The nanoparticles also showed a high in vitro affinity to bone components, including HAp and bone slices, and their affinity to HAp was not disrupted by the presence of calcium ions or low-pH conditions, both of which promote bone resorption by activated osteoclasts. 

Very recently, protein/CH-PEP·Na conjugates were prepared via simple thermal treatment (Figure 5d), exploiting the hydrophobic attraction between proteins and cholesteryl groups [32]. It was found that CH-PEP·Na considerably increased the stability of the protein to heat due to its amphiphilicity and negative charges and that the CH-PEP·Na chains were also able to protect the complexed proteins in the presence of proteolytic cleavage, while the complex size stability was excellently maintained over one month. In addition, fluorescence-labeled bovine serum albumin/CH-PEP·Na conjugates have been shown to have a binding affinity for HAp surfaces, even in the presence of high concentrations of free albumin. 

## 5. Cellular Interaction of Polyphosphodiesters (PPDEs)

### 5.1. Cytocompatibility

**Figure 6:**
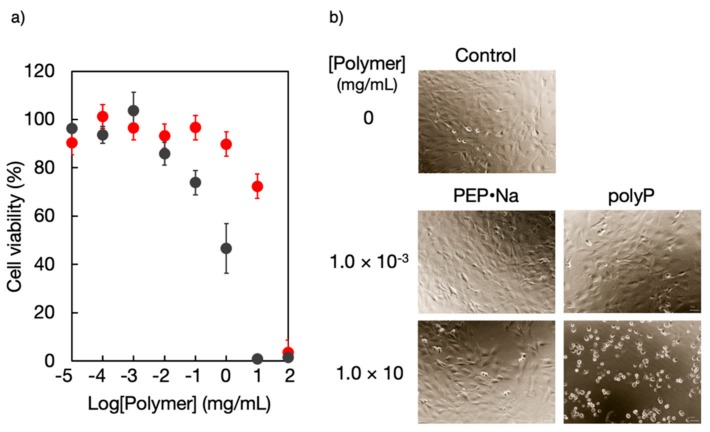
(**a**) Viability and (**b**) morphology of MC3T3-E1 cells following contact with poly(ethylene sodium phosphate) (PEP·Na) or poly(phosphate) (polyP). ●: PEP·Na; ●: polyP. Reproduced with permission from reference [33]. Copyright 2018 by The Royal Society of Chemistry.

The cytocompatibility of PPEs has been confirmed in various studies and similarly PPDEs have been shown to exhibit excellent cytocompatibility, which is greatly enhanced by sodium salt formation through their neutralization. We found that osteoblastic MC3T3-E1 cells that had been in contact with various concentrations of PEP·Na or poly(phosphate) (polyP) for 24 h exhibited a decreased viability with an increased concentration of either polymeric additive but had a higher viability with PEP·Na than with polyP, with 50% inhibition concentration (IC_50_) values of approximately 20.0 mg/mL (2.09 mM) for PEP·Na and 0.9 mg/mL (0.14 mM) for polyP (Figure 6a) [33]. In addition, the IC50 of PEP·Na is significantly higher than that of the second-generation bisphosphonate pamidronate (3.8 × 10^−2^ mg/mL) [15]. Examination of the morphologies of MC3T3-E1 cells showed that all adherent cells on a tissue culture dish exhibited a healthy spindle shape when cultured under controlled conditions in medium with no polymer additives or containing 10 mg/mL PEP·Na (75% viability), but became spherical when cultured in medium containing 10 mg/mL polyP (0% viability) (Figure 6b). Thus, PEP·Na shows superior biocompatibility compared with polyP, which is currently considered an interesting candidate polymer for bone tissue engineering. 

### 5.2. Osteoblast Differentiation

There has also been interest in the effect of PPEs on the functions of bone cells. Bone homeostasis is maintained by a balance between osteoclastic bone resorption and osteoblastic bone formation. Yang et al. [34] were the first to report that PPEs had an osteoinductive potential. They synthesized block copolymers composed of PLLA and PPE and found that osteoblast adhesion, proliferation, and function were upregulated on the copolymers compared with on PLLA. However, the relationship between the chemical structure of the copolymers and their osteoinductive potential has not yet been fully addressed. 

Nifant’ev et al. [35] also recently reported the enhancement of osteoblastic differentiation by PPDEs. They synthesized PEP via the route shown in Figure 2b and made the polysalts PEP·Na and PEP·Ca in the aqueous phase. They then pretreated cell culture dishes with aqueous solutions of PEP·Na or PEP·Ca and cultured human adipose tissue-derived stem cells on the dishes. They found that neither the sodium nor calcium PPDE salts had a toxic effect over seven days. However, the calcium PPDE salts clearly induced osteogenic differentiation of the adipose tissue-derived mesenchymal stem cells, whereas the sodium salts were inactive within the margin of experimental error.

### 5.3. Osteoclastic Differentiation

**Figure 7:**
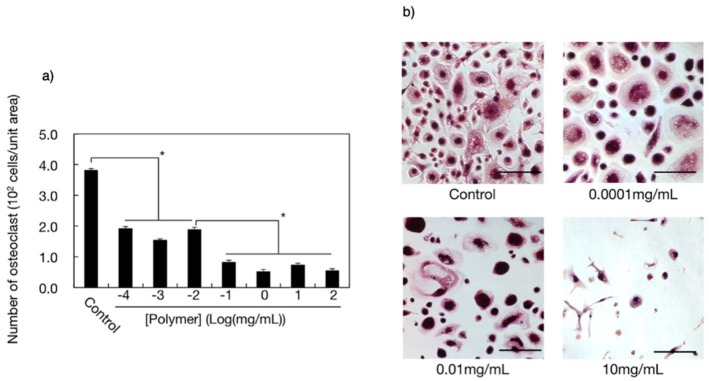
(**a**) Densities of adherent osteoclasts on bovine bone slices after incubation with poly(ethylene sodium phosphate) (PEP·Na) for 24 h (*n* = 4). Student’s t-test was performed on the samples to test the statistical significance of differences (*p < 0.005). (**b**) Optical micrographs of adherent osteoclasts on a bovine bone slice after cultivation with PEP·Na for 24 h. Scale bars represent 100 μm. Reproduced with permission from reference [36]. Copyright 2015 by Wiley Interscience.

After four weeks of osteoclast induction, cultures of mature human osteoclasts on bone slices were exposed to PEP·Na for 24 h to assess its effect on cell viability [36]. The tartrate-resistant acid phosphatase (TRAcP) activity was assessed with a leukocyte acid phosphatase kit, whereby cells with three or more nuclei were considered TRAcP-positive multinucleated cells (TPMCs) and the numbers of TPMCs were counted from microscopic images using Image J. This analysis showed that the addition of PEP·Na to the cultivation medium significantly reduced osteoclast adhesion on bovine bone slices (Figure 7a). 

Optical micrographs of osteoclasts (including giant cells) that had been incubated with different concentrations of PEP·Na and then stained for TRAcP are shown in Figure 7b. In the absence of PEP·Na, many cells adhered to the bone slice and large, well-distributed cells were observed. When a low concentration of PEP·Na (1 × 10^−4^ mg/mL) was added to the cultivation medium, an increased number of mature osteoclasts remained adhered but many smaller adherent cells were also observed. However, further increases in the concentration of PEP·Na resulted in a significant decrease in the number of adherent cells as well as a reduction in their size. Although the mechanism behind the interaction between PEP·Na and osteoclasts has not yet been explored, these findings indicate that PEP·Na and the degraded products affect the molecular metabolism of osteoclasts. 

## 6. In Vivo Bone Targeting

**Figure 8:**
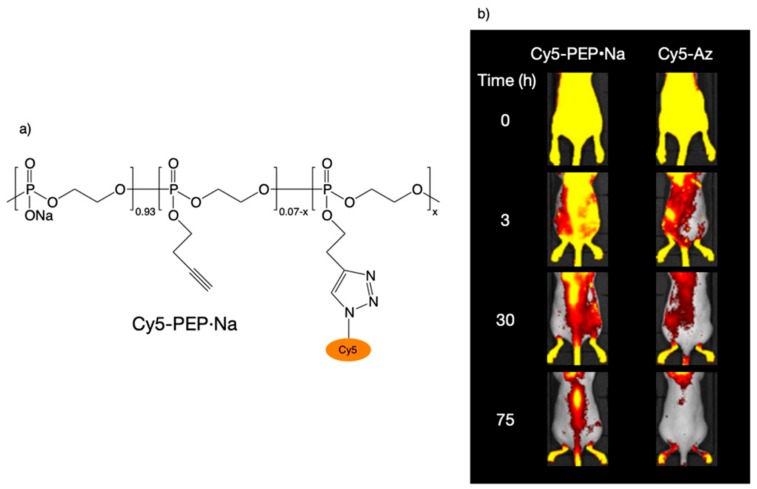
(**a**) Chemical structure of fluorescence-labeled poly(ethylene sodium phosphate) (Cy5-PEP·Na) and (**b**) in vivo fluorescence imaging of ICR mice at 0, 3, 30, and 75 h after the intravenous injection of Cy5-PEP·Na or Cy5-Az. Reproduced with permission from reference [33]. Copyright 2018 by The Royal Society of Chemistry.

To track the biodistribution of PEP·Na in vivo, fluorescence-labeled PEP·Na (Cy5-PEP·Na) was synthesized (Figure 8a) and 100 μL of 100 mg/mL Cy5-PEP·Na or 100 µg/mL Cy5-Az was injected into the tail veins of ICR mice [33,37]. Each of the sample solutions had nearly identical fluorescence spectra, and although the concentration of Cy5-PEP·Na was relatively high, the polymer solution was diluted immediately after it mixed with the blood. Immediately after intravenous injection, strong fluorescence signals were observed from the whole body of each mouse (Figure 8b), suggesting that both Cy5-PEP·Na and Cy5-Az rapidly spread throughout the body via the bloodstream. However, the total radiant efficiencies of the spines of Cy5-Az-treated mice returned to the same levels as before the injections after 30 h, becoming almost extinguished, whereas fluorescence signals from the spines of Cy5-PEP·Na-treated mice were still observed 75 h after intravenous injection (Figure 8b). Because the observations were made in the dorsal position, the fluorescence signals from the bone located near the surface were significant.

## 7. Conclusions

The purpose of this review was to describe current research on PPDEs. The most popular process for preparing PPDEs is side-chain deprotection of polyphosphotriesters, and several superior deprotection processes have been proposed that do not cause degradation of the polymer backbone. The mineral affinity of PPEs depends on the amount of ionized phosphodiester units they contain, with fully ionized PPEs, such as PEP·Na, showing a high affinity to bone both in vitro and in vivo. Although the mechanism behind the effect of PPDEs on the functions of bone cells has not yet been clarified, it has been reported that they upregulate osteoblastic activity and downregulate osteoclastic differentiation, which is similar to the effects of anabolic drugs that are used to treat osteoporosis. Furthermore, PPDEs would also have the ability to form conjugates with therapeutic proteins or drugs. Therefore, PPDEs may represent interesting macromolecular pharmacology platforms for bone treatment.
